# Effect of benralizumab treatment on the airway microbiome in COPD

**DOI:** 10.1183/23120541.00802-2024

**Published:** 2025-04-07

**Authors:** Koirobi Haldar, Vijay Mistry, Mathew Richardson, Corinne Hamblet, Maria Jison, Michael R. Barer, Christopher McCrae, Christopher E. Brightling

**Affiliations:** 1 Institute for Lung Health, NIHR, BRC, Department of Respiratory Sciences, College of Life Sciences, University of Leicester, Leicester, UK; 2 Translational Science and Experimental Medicine, Early Respiratory and Immunology, BioPharmaceuticals R&D, AstraZeneca, Gothenburg, Sweden; 3 Late-stage Respiratory and Immunology, BioPharmaceuticals R&D, AstraZeneca, Gaithersburg, MD, USA; 4 Department of Respiratory Sciences, College of Life Sciences, University of Leicester, Leicester, UK; 5 Translational Science and Experimental Medicine, Early Respiratory and Immunology, BioPharmaceuticals R&D, AstraZeneca, Gaithersburg, MD, USA

## Abstract

**Background:**

One-third of patients with COPD have an eosinophilic inflammatory phenotype. Benralizumab is an afucosylated humanised monoclonal antibody that targets the interleukin-5 receptor α subunit, leading to rapid and near-complete eosinophil depletion *via* antibody-dependent cellular cytotoxicity. We hypothesised that benralizumab-targeted immune modulation could have an impact on the airway microbiome in COPD. The objective of the present study was to investigate the effect of benralizumab treatment on inflammation and the sputum microbiome in COPD.

**Methods:**

Sputum samples from 94 COPD patients enrolled to the GALATHEA trial (NCT02138916) and randomised to receive placebo (33), benralizumab at 100 mg (29) or 30 mg (32) over 52 weeks were analysed at baseline, week 24 and at end of treatment (week 56). Sputum microbiota taxonomic profiles and diversity indices, generated from paired-end Illumina sequencing targeting the 16S rRNA gene, were used for comparative analyses. Linear mixed model analyses were applied to blood and sputum cell counts and eosinophil mediators for within- and between-treatment group analyses.

**Results:**

Participants treated with 100 and 30 mg benralizumab, respectively, showed a significant reduction from baseline in both blood and sputum eosinophil counts (blood: p=1.2e-10 and p=8.8e-10; sputum p=0.03 and p=0.004) and eosinophil-derived serum mediators (eosinophil cationic protein: p<3e-09 and p<2e-08; eosinophil-derived neurotoxin: p<8e-12 and p<2e-09). No significant changes in the composition or diversity of the sputum microbiome were observed.

**Conclusions:**

In this study, the airway microbiome measured in sputum was unaffected by a targeted reduction of eosinophilic inflammation with benralizumab treatment.

## Shareable abstract

**The sputum microbiota in benralizumab-treated COPD patients remains unaffected despite a targeted reduction in the eosinophil counts and associated inflammatory mediators, suggesting no immune-modulatory effect of treatment on the airway microbiome** https://bit.ly/40hd89B

## Introduction

COPD is a complex, heterogeneous and progressive disease characterised by a spectrum of chronic airway inflammation, including both neutrophil and eosinophil predominant patterns [1]. The airway microbiome has also been shown to play an important role in disease progression and exacerbations, with distinct microbiome profiles and host–microbe interactions associating with neutrophilic and eosinophilic inflammatory phenotypes [2].

Approximately one-third of stable COPD cases exhibit a type 2 inflammatory phenotype with elevated blood and sputum eosinophil counts, which is associated with increased exacerbation frequency and hospitalisation rates [3, 4]. Eosinophil levels serve as a biomarker to guide inhaled corticosteroid (ICS) therapy, which is efficacious in this group to reduce exacerbations [5, 6]. However, ICS use carries an increased risk of infections, particularly pneumonia in COPD [7, 8]. These broad-spectrum anti-inflammatory agents can suppress the local immune system, leading to alterations in the airway microbiome and potentially pathogenic bacterial proliferation [7, 9]. However, it is unclear whether steroid-related microbial dysbiosis is caused by the attenuation of eosinophils or the altered function of other immune pathways.

In contrast to the broad immunosuppressive effects of corticosteroids, monoclonal antibody-based biologic therapies offer an alternative approach that specifically target immune pathways or cytokines involved in eosinophilic inflammatory responses. Moreover, it has been suggested that elevated levels of airway interleukin (IL)-5 might be associated with reduced efficacy of corticosteroid-mediated eosinophil apoptosis in severe eosinophilic asthma [10]. IL-5 is a major cytokine involved in eosinophil-mediated inflamma­tion. It promotes the maturation, migration, activation, survival and effector functions of eosinophils. Benralizumab, an afucosylated monoclonal antibody, dampens eosinophilic inflammation through a dual mechanism, blocking the IL-5 receptor on eosinophils and recruiting natural killer cells for antibody-dependent cell-mediated cytotoxicity [11]. Although it has been suggested that anti-IL-5 therapy might be effective in reducing the exacerbation risk in COPD, little is known of the impact of this treatment on the airway microbiome [12]. The eosinophil and its inflammatory mediators have been shown to possess antibacterial properties that may influence microbial composition, with depletion causing clinically important microbiome changes [13, 14].

This study aimed to investigate the impact of benralizumab treatment on the airway microbiome in patients with moderate to severe COPD, focusing on the potential role of benralizumab-mediated immune modulation. The specific objectives of the study were to characterise the composition of the airway microbiome in COPD patients before and after benralizumab treatment, to assess how benralizumab treatment influences the airway microbiome through its effects on immune cell populations (particularly eosinophils), and to determine if changes in the airway microbiome following benralizumab treatment correlate with clinical outcomes in patients with COPD.

## Methods

### Study design and participants

The studied subjects and samples are a subset of the previously completed prospective, randomised, double-blind phase 3 trial of benralizumab in COPD (GALATHEA: NCT02138916). The trial enrolled moderate to severe COPD patients who were frequent exacerbators despite receiving guideline-based standard inhaled therapy. Participants were recruited at a 2:1 ratio according to whether their baseline blood eosinophil count was ≥220 or <220 per cubic millimetre, respectively. Participants were randomised to receive 1-year treatment with benralizumab (30 or 100 mg every 8 weeks, initial three doses every 4 weeks) or placebo. Further information on the enrolment criteria and clinical metadata collection are detailed in the original publication [15]. Induced sputum samples were collected for microbiological analysis at multiple time-points during the clinical trial. For this study, patients with an existing baseline sputum sample were selected and, whenever available, intermediate week 24 (V11) and post-treatment week 56 (V19) samples were included (supplementary figure 1). Corresponding clinical metadata on lung function and health quality assessment scores, blood and sputum cell count, and sputum and serum inflammatory markers were recorded. A microscopy-based differential cell count of up to 400 cells was performed on methanol-fixed cells stained with the RapiDiff II stain to evaluate the presence of neutrophils, macrophages, eosinophils, bronchial epithelial cell and lymphocytes in sputum. Immunoassays were performed for quantifying eosinophil-derived neurotoxin (EDN) (ELISA, Alpco Diagnostics) and eosinophil cationic protein (ECP) (ImmunoCAP, Thermo Fisher scientific). Independent ethics committees of the trial centres or central institutional review boards approved the trial protocols and the trials were conducted in accordance with the principles of the Declaration of Helsinki. All patients provided written informed consent [15].

### Sputum sample processing and DNA extraction

To minimise oral bacterial contamination, sputum plugs were separated from salivary contents in the analysed samples and stored at −80 °C until they were processed for DNA extraction. Bacterial genomic DNA was extracted from the homogenised (0.1% dithiothreitol) plugs using a lysozyme-based lysis procedure from the Qiagen DNA Mini kit (Qiagen, CA, USA) as per the manufacturer's protocol. Of the 284 sputum DNA samples from 122 participants, after excluding samples from participants without a baseline sputum DNA, only 231 samples from 105 participants qualified for microbial sequencing (supplementary figure 2). Bacterial loads were enumerated, based on the abundance of 16S ribosomal subunit encoding genes, by quantitative PCR (qPCR) on ThermoFisher Quantstudio 5 apparatus (Life Technologies, Paisley, UK) using previously described primers and cycling conditions [16].

### Amplicon library generation and sequencing

For sequencing, amplicon libraries targeting the V4 hypervariable region of the 16S rRNA gene were generated using 28 PCR cycles with primers 515F (5′-GTGCCAGCMGCCGCGGTAA-3′) and 806R (5′-GGACTACHVGGGTWTCTAAT-3′), including Illumina sequencing adapters and a 12 bp Golay barcode sequence attached to the forward primer. Paired-end sequencing (250 bp) was performed on the Illumina HiSeq2500 platform. Sequencing runs included commercial mock community DNA (ZymoBIOMICS microbial DNA standard) as a positive control, two DNA extraction negative controls (each batch of DNA extraction included a DNA extraction negative control and a single pooled aliquot was prepared from all of these controls for sequencing) and PCR negative controls for reagent contamination checks. The PCR negative controls did not produce any reads and the DNA extraction negative controls produced <5000 (79–4939) raw reads. Sequencing was conducted at the Centre of Genomic Research (CGR) hub at Liverpool university.

### Microbiome analysis

The QIIME (Quantitative Insights Into Microbial Ecology) v1.9.1 pipeline was used for processing of the demultiplexed reads received from the CGR post-Illumina adapter sequence trimming (Cutadapt v1.2.1) and filtering out reads shorter than 20 bp Sickle (v1.2) for subsequent analysis [17]. Paired-end sequences were merged using fastq-join (https://github.com/ExpressionAnalysis/ea-utils/blob/wiki/FastqJoin.md) (minimum overlap of 10 bp) [18]. Joined sequences were further filtered for Phred score (≥20) and potential contaminants/chimeras were removed using UCHIME [18]. 15 samples exhibited no visible amplicon library bands and were excluded due to potential spurious reads (median: 4313). operational taxonomic units (OTUs) were defined at 97% sequence identity using the Greengenes database (v13_8) and assigned taxonomy with the Ribosomal Database Project classifier [19]. Based on rarefaction curves and the maximum read depth of failed samples, a sequencing depth of 31 750 reads was chosen for COPD sample normalisation, resulting in 207 samples from 94 patients for microbiome diversity analysis (supplementary figure 2). Alpha diversity indices (Chao1 richness, Shannon, equitability and phylogenetic diversity) were calculated to assess within-sample OTU richness and evenness. Beta diversity between samples was assessed using weighted UniFrac distance [20] and visualised with principal coordinate analysis plots. Differences in microbiome composition between treatment groups were statistically evaluated using PERMANOVA with weighted UniFrac distance as the input. Gammaproteobacteria to Firmicutes (GP:F) ratio has been shown to be an important microbiome marker in characterising eosinophilic and neutrophilic inflammatory phenotype [21] and a higher Firmicutes to Bacteroidetes (F:B) ratio was observed in baseline samples with ≥3% *versus* <3% sputum eosinophil counts in this study [22]. Therefore, these two ratios were used as univariate microbiome parameters for performing association analysis with clinical parameters and biomarkers. The microbiome ratios were deduced by log 2 transformation of the ratio of proportion of the sequences represented by these groups in a sample. The sequence data are deposited at the National Center for Biotechnology Information Sequence Read Archive (SRA accession: PRJNA1114681) (www.ncbi.nlm.nih.gov/bioproject/PRJNA1114681/).

### Statistical analysis

Summary statistics of clinical data and significance analyses were performed using R (v4.2.3). Descriptive statistics were presented as mean±sd median (interquartile range) and counts (%) for parametric, nonparametric and nominal data, respectively. Between-group significance tests of two or greater than two groups, respectively, were performed by applying the t-test and ANOVA for parametric data and the Mann–Whitney and Kruskal–Wallis tests for nonparametric data. For categorical data, the Chi-square test was used. Spearman correlation matrix analysis was performed to establish any association between the microbiome and clinical parameters. Multiple correlation comparisons were adjusted using the Holm–Bonferroni method. Linear mixed model analyses were performed on clinical variables to account for missing data. The Box–Cox transformation was applied to non-normal data to improve the accuracy of predictions made using linear mixed models. For within-treatment group analysis, baseline samples were set as the reference category and for between-treatment group analysis, baseline visit and placebo group were both used as reference categories. For the within-treatment group linear mixed models, the p-value for the visit represents the combined significance for all visit coefficients derived from performing a likelihood ratio test of the visit mixed model to the null model within each treatment group.

## Results

**FIGURE 1:**
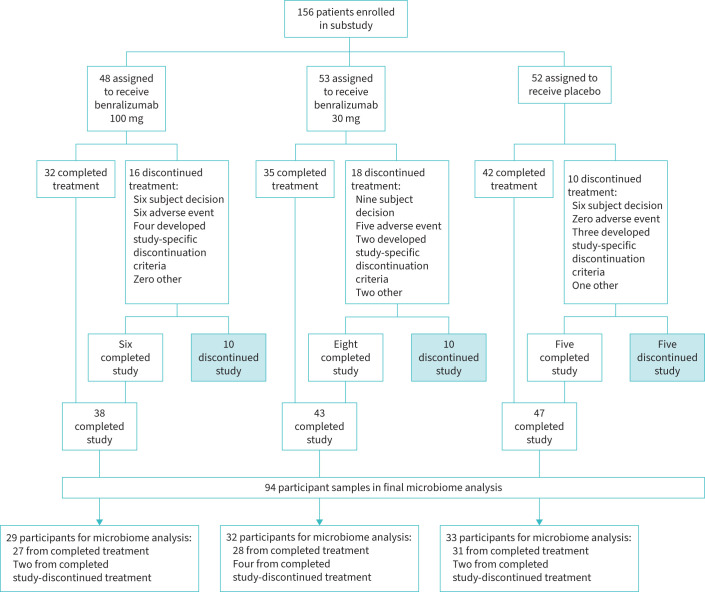
Schematic flow diagram of the GALATHEA trail patient recruitment process. Out of 19 participants who had completed study but discontinued treatment only 13 had sample collected for microbiome. Out of these, only eight participant samples were included in the final microbiome analysis as two participants did not have baseline samples available and the other three participant baseline samples failed to produce amplicon libraries. Therefore, these were excluded from the analysis.

The number of participants enrolled and completing the study in each of the three treatment arms for the GALATHEA study are as shown in figure 1. Eight participants included in the microbiome analysis had discontinued treatment but completed the study; all samples analysed here, with the exception of two (V19) samples from the benralizumab 30 mg group, were obtained before the discontinuation of the treatment. As there was no difference in the microbiome composition with or without excluding these samples, these were included in the final analysis (supplementary figure 3).

**FIGURE 2:**
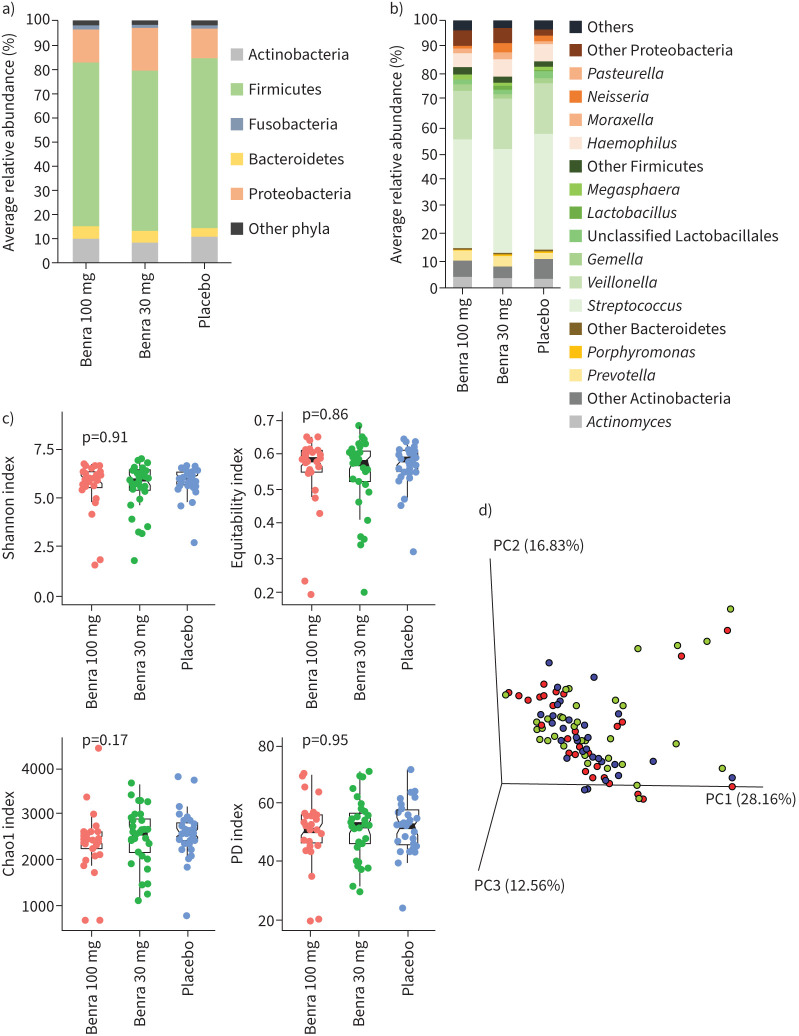
No difference in baseline microbiota between the treatment groups. a and b) The taxonomic distribution of the microbiota at phylum and genus level. c) Alpha diversity metrics. d) Principal coordinate analysis beta diversity plot based on the weighted UniFrac distance. Benra: benralizumab; PC: principal component; PD: phylogenetic diversity.

### Baseline clinical characteristics and microbiota composition

94 participants were included in the microbiome analysis, with 29, 32 and 33 baseline samples from the benralizumab 100 mg and 30 mg and placebo groups, respectively. Clinical and inflammatory characteristics of the three treatment allocation groups were well matched at baseline (table 1). All groups had elevated sputum and blood eosinophil counts compared to the general COPD population [3].

**TABLE 1:** Baseline clinical characteristics

Characteristic	Benra 100 mg	Benra 30 mg	Placebo	p-value
**n**	29	32	33	
**Age, years**	63.83 (8.32)	67.69 (7.95)	65.79 (7.98)	0.18
**Male, n(%)**	17 (58.6)	21 (65.6)	20 (60.6)	0.84
**BMI, kg·m^−2^**	31.77 (7.51)	28.14 (5.97)	29.97 (7.48)	0.14
**Former smokers (%)**	20 (69.0)	25 (78.1)	23 (69.7)	0.67
**Pack-years**	49.97 (25.88)	49.94 (19.24)	45.67 (23.16)	0.69
**Exacerbations in previous year**	2.00 (2.00–3.00)	2.00 (2.00–3.00)	3.00 (2.00–4.00)	0.42
**mMRC dyspnoea scale, %**				0.4
1	8 (27.6)	7 (21.9)	8 (24.2)	
2	9 (31.0)	13 (40.6)	6 (18.2)	
3	10 (34.5)	7 (21.9)	14 (42.4)	
4	2 (6.9)	5 (15.6)	5 (15.2)	
**SGRQ score**	57.54 (15.86)	53.89 (15.37)	55.56 (15.79)	0.66
**CAT score**	22.76 (5.81)	20.38 (7.01)	22.06 (7.29)	0.37
**FEV_1_, L**	1.04 (0.35)	0.93 (0.37)	1.02 (0.45)	0.51
**FEV_1_, % pred**	36.92 (12.23)	34.51 (13.62)	35.58 (13.31)	0.77
**FVC, L**	2.62 (0.71)	2.55 (0.90)	2.61 (0.90)	0.94
**FVC, % pred**	71.48 (17.32)	71.65 (20.63)	70.18 (17.81)	0.94
**FEV_1_/FVC**	40.90 (11.79)	37.84 (11.79)	39.30 (10.30)	0.58
**Sputum eosinophils, %**	3.00 (1.50–8.00)	6.75 (2.00–13.75)	3.12 (1.00–6.94)	0.42
**Sputum neutrophils, %**	73.00 (54.25–85.00)	71.75 (56.88–84.12)	71.88 (63.56–83.94)	0.98
**Sputum TCC, 10^6^·g^−1^**	3.36 (1.04–5.94)	4.14 (2.17–7.02)	2.68 (1.06–8.79)	0.62
**Blood eosinophils, ×10^9^·L^−1^**	0.33 (0.18–0.40)	0.23 (0.18–0.41)	0.28 (0.19–0.35)	0.68
**Blood neutrophils, ×10^9^·L^−1^**	4.33 (4.02–5.32)	5.50 (4.72–6.62)	4.97 (4.03–6.02)	0.14
**Lung condition (%)**				0.86
** **Chronic bronchitis	8 (27.6)	8 (25.0)	11 (33.3)	
** **Emphysema	8 (27.6)	6 (18.8)	6 (18.2)	
Both	8 (27.6)	14 (43.8)	11 (33.3)	
None	5 (17.2)	4 (12.5)	5 (15.2)	
**Total bacterial load^#^**	9.08 (0.58)	8.89 (0.42)	9.06 (0.66)	0.34

^#^: log 10 transformation. In these cases, the ANOVA (including for geometric mean), Kruskal–Wallis and chi-squared statistical tests were applied. Data is shown as mean (sd) for parametric and median (interquartile range) for nonparametric data unless otherwise stated. BMI: body mass index; CAT: COPD Assessment Test; FEV_1_: forced expiratory volume in 1 s; FVC: forced vital capacity; mMRC: modified Medical Research Council; SGRQ: St. George's Respiratory Questionnaire; TCC: total cell count.

Composition of the sputum microbiome was also similar between the groups. Firmicutes was the dominant phylum across all three treatment groups, with *Streptococcus* and *Veillonella* at genus level contributing ∼75% of the abundance (figures 2a and 2b). *Haemophilus* and *Moraxella* were the dominant genera representing the Proteobacteria phylum (figure 2b). There was no difference in either alpha or beta diversity measure between the treatment groups (figures 2c and 2d). There was no difference in either GP:F or F:B ratio between the treatment groups. At baseline there was no association between any of the microbial constituents with clinical parameters (supplementary figure 4). Stratifying samples based on baseline blood eosinophil groups into low (<220 cells·µL^−1^), mid (220–299 cells·µL^−1^) and high (≥300 cells·µL^−1^) showed no difference in the microbiome diversity metrics between these groups (Supplementary fig 5).

### Effect of benralizumab treatment on eosinophil markers and clinical parameters

Participants treated with benralizumab at both 30 mg and 100 mg dosages showed a significant depletion from baseline in % sputum eosinophil counts (p=0.004 and p=0.03) and blood eosinophil counts (p=8.8E-10 and p=1.2E-10), respectively (table 2). This cellular depletion with benralizumab treatment was associated with a corresponding significant reduction in serum levels of eosinophil-derived products, including EDN (p=2e-09 and p=7.7e-12) and ECP (p=1.8e-08 and p=3.3e-09) at both 30 mg and 100 mg doses, respectively. Depletion of eosinophil and its related proteins at individual time-points at both dosages are summarised in supplementary table 1. In sputum, a significant reduction in EDN was observed only in the 30 mg group (p=0.02). Additionally, the 100 mg dose was associated with a decrease in symptom scores (p=0.04) (table 2). Samples from placebo-treated participants also showed a significant decrease in blood eosinophil count and serum ECP from baseline but at a much lower magnitude compared to the benralizumab-treated group (supplementary table 1).

**TABLE 2:** Benralizumab (benra)-treated visits showed a higher reduction in their eosinophil-related markers

Benra 100 mg	Baseline	Week 24	Week 56	p-value
	n=29	n=16	n=13	
**Blood eosinophils, ×10^9^·L^−1^**	0.33 (0.18–0.40)	0.00 (0.00–0.00)	0.00 (0.00–0.00)	1.2e-10
**Sputum eosinophils, %**	3.00 (1.50–8.00)	0.00 (0.00–0.25)	0.00 (0.00–0.00)	0.03
**Serum EDN, ng·mL^−1^**	55.21 (33.53–99.94)	7.83 (3.75–12.91)	4.45 (4.44–7.95)	7.7e-12
**Serum ECP, ng·mL^−1^**	14.03 (8.09–26.09)	4.14 (2.00–11.28)	4.48 (2.02–9.72)	3.3e-09
**Sputum EDN, ng·mL^−1^**	129.34 (58.65–266.28)	48.51 (15.14–102.91)	50.77 (30.54–97.93)	0.05
**Blood neutrophils, ×10^9^·L^−1^**	4.33 (4.02–5.32)	4.76 (3.81–5.85)	4.66 (3.91–5.06)	0.57
**Sputum neutrophils, %**	73.00 (54.25–85.00)	87.25 (70.50–91.00)	79.00 (40.00–89.62)	0.30
**Sputum TCC, 10^6^·g^−1^**	3.36 (1.04–5.94)	1.71 (1.14–4.15)	1.46 (1.03–1.99)	0.73
**CA** **T score**	22.76 (5.81)	21.06 (6.35)	19.08 (6.37)	0.04
**SGRQ score**	57.54 (15.86)	56.75 (15.48)	51.54 (14.29)	0.27
**FEV_1_, L**	1.04 (0.35)	1.07 (0.38)	0.99 (0.37)	0.97
**FEV_1_, % pred**	36.92 (12.23)	36.03 (11.23)	35.08 (10.92)	0.98
**FVC, L**	2.62 (0.71)	2.70 (0.74)	2.63 (0.82)	0.96
**FVC, % pred**	71.48 (17.32)	71.00 (19.82)	70.45 (18.91)	0.94
**FEV_1_/FVC**	40.90 (11.79)	40.62 (10.80)	39.77 (13.21)	0.70

Benra 30 mg	n=32	n=20	n=19	
**Blood eosinophils, ×10^9^·L^−1^**	0.23 (0.18–0.41)	0.00 (0.00–0.01)	0.00 (0.00–0.01)	8.8e-10
**Sputum eosinophils, %**	6.75 (2.00–13.75)	0.00 (0.00–0.00)	0.00 (0.00–0.00)	0.004
**Serum EDN, ng·mL^−1^**	35.75 (31.35–50.61)	5.93 (3.37–8.23)	4.68 (3.12–11.72)	2e-09
**Serum ECP, ng·mL^−1^**	16.16 (6.56–24.20)	3.33 (2.00–6.60)	2.72 (2.49–3.97)	1.8e-08
**Sputum EDN, ng·mL^−1^**	172.38 (68.05–210.81)	43.31 (11.65–82.97)	46.35 (45.08–50.03)	0.02
**Blood neutrophils, ×10^9^·L^−1^**	5.50 (4.72–6.62)	5.30 (4.65–7.38)	6.11 (5.46–6.70)	0.41
**Sputum neutrophils, %**	71.75 (56.88–84.12)	83.50 (66.50–90.25)	83.75 (69.25–92.00)	0.13
**Sputum TCC, 10^6^·g^−1^**	4.15 (2.17–7.03)	2.32 (1.28–13.4)	2.65 (0.93–7.69)	0.51
**CAT score**	20.38 (7.01)	20.80 (5.93)	20.53 (7.34)	0.82
**SGRQ score**	53.89 (15.37)	52.53 (15.91)	55.73 (21.04)	0.40
**FEV_1_, L**	0.93 (0.37)	0.86 (0.35)	0.81 (0.31)	0.68
**FEV_1_, % pred**	34.51 (13.62)	30.87 (12.80)	28.78 (10.53)	0.64
**FVC, L**	2.55 (0.90)	2.43 (0.76)	2.36 (0.82)	0.85
**FVC, % pred**	71.65 (20.63)	66.44 (18.11)	63.53 (17.77)	0.86
**FEV_1_/FVC**	37.84 (11.79)	36.10 (11.61)	35.42 (9.69)	0.38

Placebo	n=33	n=26	n=19	
**Blood eosinophils, ×10^9^·L^−1^**	0.28 (0.19–0.35)	0.21 (0.13–0.28)	0.25 (0.15–0.31)	0.04
**Sputum eosinophils, %**	3.12 (1.00–6.94)	2.00 (0.62–4.00)	1.62 (0.69–5.31)	0.83
**Serum EDN, ng·mL^−1^**	37.41 (23.30–59.12)	28.80 (20.87–40.24)	28.25 (24.49–35.24)	0.02
**Serum ECP, ng·mL^−1^**	18.54 (9.01–29.25)	12.01 (5.71–21.47)	14.09 (7.28–26.92)	0.05
**Sputum EDN, ng·mL^−1^**	117.71 (27.47–250.89)	86.85 (22.98–214.84)	176.40 (51.23–242.89)	0.359
**Blood neutrophils, ×10^9^·L^−1^**	4.97 (4.03–6.02)	5.34 (4.03–5.92)	5.04 (3.72–6.96)	0.81
**Sputum neutrophils, %**	71.88 (63.56–83.94)	75.75 (49.00–84.12)	73.88 (59.19–79.50)	0.50
**Sputum TCC, 10^6^·g^−1^**	2.68 (1.06–8.79)	2.91 (0.54–7.97)	2.10 (0.88–4.21)	0.25
**CAT score**	22.06 (7.29)	21.80 (5.62)	21.84 (5.46)	0.92
**SGRQ score**	55.56 (15.79)	52.26 (11.94)	51.87 (14.09)	0.42
**FEV_1_, L**	1.02 (0.45)	1.02 (0.35)	1.11 (0.57)	0.29
**FEV_1_, % pred**	35.58 (13.31)	35.79 (11.52)	36.94 (15.88)	0.42
**FVC, L**	2.61 (0.90)	2.70 (0.69)	2.78 (0.76)	0.36
**FVC, % pred**	70.18 (17.81)	72.84 (13.89)	72.97 (13.21)	0.30
**FEV_1_/FVC**	39.30 (10.30)	38.24 (11.13)	38.53 (13.84)	0.60

Data is shown as mean (sd) and median (interquartile range). A linear mixed model was used to assess within-treatment and between-visit differences. p-value for visit represents the combined significance for all visit coefficients (chi square test statistic). BMI: body mass index; CAT: COPD Assessment Test; ECP: eosinophil cationic protein; EDN: eosinophil-derived neurotoxin; FEV_1_: forced expiratory volume in 1 s; FVC: forced vital capacity; SGRQ: St. George's Respiratory Questionnaire; TCC: total cell count.

Between-treatment group analysis at week 24 and week 56, respectively, showed significantly lower blood eosinophil counts (p=2.5e-05 and p=1.8e-05), sputum eosinophils (p=0.04 and p=0.04) and serum EDN (p=1.1e-10 and p=1.7e-03) and ECP (p=3.7E-04 and p=5.3e-05) with benralizumab 30 mg compared to placebo (supplementary figure 6). Benralizumab 100 mg also showed significantly lower blood eosinophil counts (p=2.1e-04 and p=1.9e-04) and serum markers (EDN p=1.1e-11 and ECP p=3.9e-04 at week 24) at both time-points, with an additional symptom score reduction (p=0.03) at week 56 compared to placebo (supplementary figure 6).

### Effect of benralizumab treatment on the airway microbiome

**FIGURE 3:**
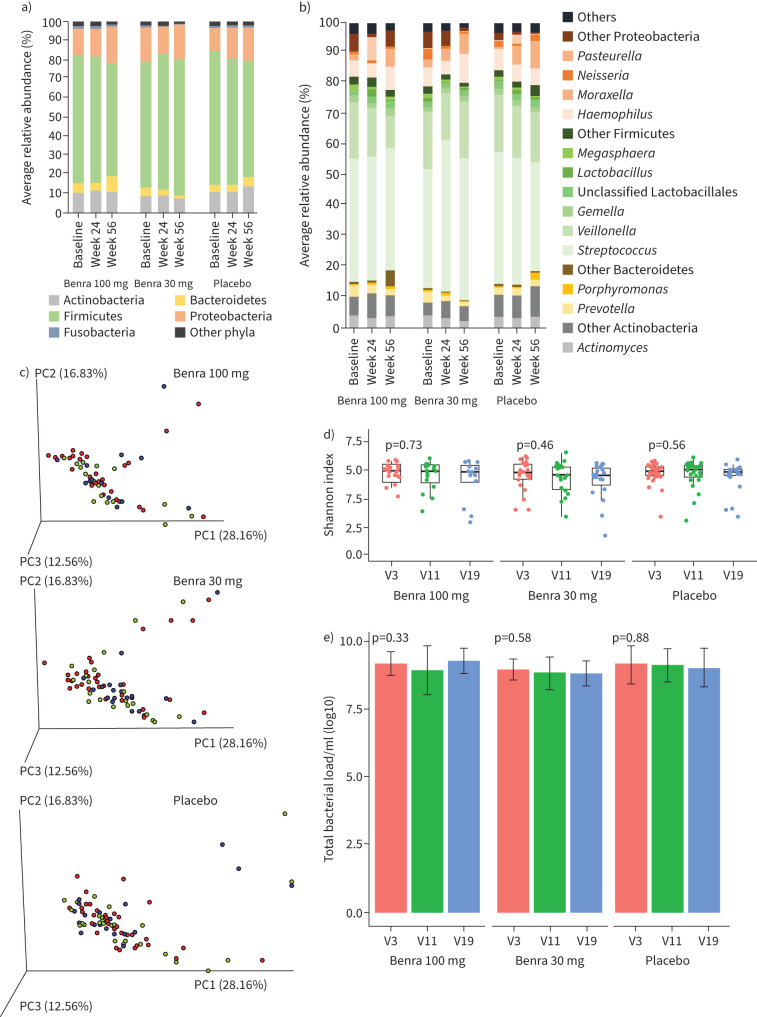
No change in microbiome across visit in the benralizumab (benra)-treated group**.** a and b) Taxonomic distribution at phylum and genus level of the microbiota. c) Principal coordinate analysis beta diversity plot based on the weighted UniFrac distance in each treatment group. d) Alpha diversity metrics. e) Total bacterial load within each treatment group across the visit time-points. PC: principal component; V: visit.

No significant difference was observed in the taxonomic distribution frequency, alpha or beta diversity indexes within the benralizumab-treated population (figures 3a, b, c and d). There was no change in the total bacterial load within the benralizumab treated group (figure 3e). Furthermore, there was no association of baseline microbiome parameters with changes in sputum and blood counts, lung function, symptom scores or inflammatory markers in benralizumab-treated participants (supplementary figure 7a). No association between change in microbiome parameters and clinical variables was observed (supplementary figure 7b).

## Discussion

Our study is the first to examine the impact of benralizumab-mediated immune modulation on the airway microbiome in COPD patients. Benralizumab treatment at both 30 and 100 mg significantly reduced eosinophil counts, improved symptom scores and decreased eosinophilic mediators from baseline compared to placebo. However, these reductions in immune markers were not accompanied by significant alterations in overall airway microbiome composition or bacterial load.

These findings align with previous observations from a phase 2a study that reported no significant changes in bacterial abundances following benralizumab treatment [16]. In that study, COPD patients showed a small decrease in *Streptococcus pneumoniae* and total bacterial load and no change in *Haemophilus influenzae* load, compared to placebo. The study also reported *in vitro* experiments demonstrating no direct bactericidal effect of eosinophils on key COPD pathogens including *S. pneumoniae* and *H. influenzae*, suggesting that the observed *in vivo* response may not be mediated solely by eosinophil–bacteria interactions [16]. Similarly, in the current study, we found that a decrease in eosinophils and their inflammatory mediators (EDN and ECP), which play a key role in eosinophil-related antibacterial activities [14], was not associated with changes to the microbiome. A similar finding has also been reported with mepolizumab (an anti-IL-5 monoclonal antibody) treatment in asthma, where no change in sputum bacterial abundances was observed pre- and post-treatment [23]. Together, these studies provide concordant evidence that the airway microbiome is unaffected by treatment with targeted anti-eosinophil therapies in chronic airways diseases. This further suggests that steroid-induced dysbiosis is likely to be caused by broader immunosuppressive effects, upon other immune and structural cells, unrelated to eosinophilic immune pathways.

Key strengths of this study were that it was performed in a well-characterised cohort of COPD patients that had comparable between-group baseline clinical and microbiome characteristics and underwent systematic longitudinal assessment and sample collection. Although the original trial (GALATHEA: NCT02138916) was adequately powered to address its primary outcomes [15], this study, based on a smaller subset of the original study, was constrained in exploring any subgroup-specific effects due to a reduced sample size. The study was also limited by selection bias as we could not investigate whether the influence of benralizumab treatment on the microbiome would differ between noneosinophilic and eosinophilic inflammatory phenotypes of COPD. Viral and fungal communities have also been shown to play a role in COPD [24]; however, due to a limited sample volume, we could not examine the effect of biologics on these communities. Further, larger studies in more diverse cohorts are required to address these questions. However, our data suggests that treatment with anti-IL-5/anti-IL-5Rα biologic agents leads to significant downregulation of eosinophilic inflammation in COPD, without discernible perturbation of the airway microbiome.

## Conclusions

In conclusion, benralizumab treatment effectively reduced systemic and airway eosinophilic inflammation and improved symptoms in moderate to severe COPD, but did not affect the composition of the airway microbiome.

## Supplementary material

10.1183/23120541.00802-2024.Supp1 **Please note:** supplementary material is not edited by the Editorial Office, and is uploaded as it has been supplied by the author.
Supplementary material 00802-2024.SUPPLEMENT
