# Supplementary material for: Effect of benralizumab treatment on the airway microbiome in COPD
Source: ERJ Open Res. 2025 Apr 7;11(2):00802-2024. doi: 10.1183/23120541.00802-2024 (PMC11973710; doi:10.1183/23120541.00802-2024)
Supplement: Supplementary file 1 [file 00802-2024.SUPPLEMENT.pdf]

Supplementary Figures and Tables

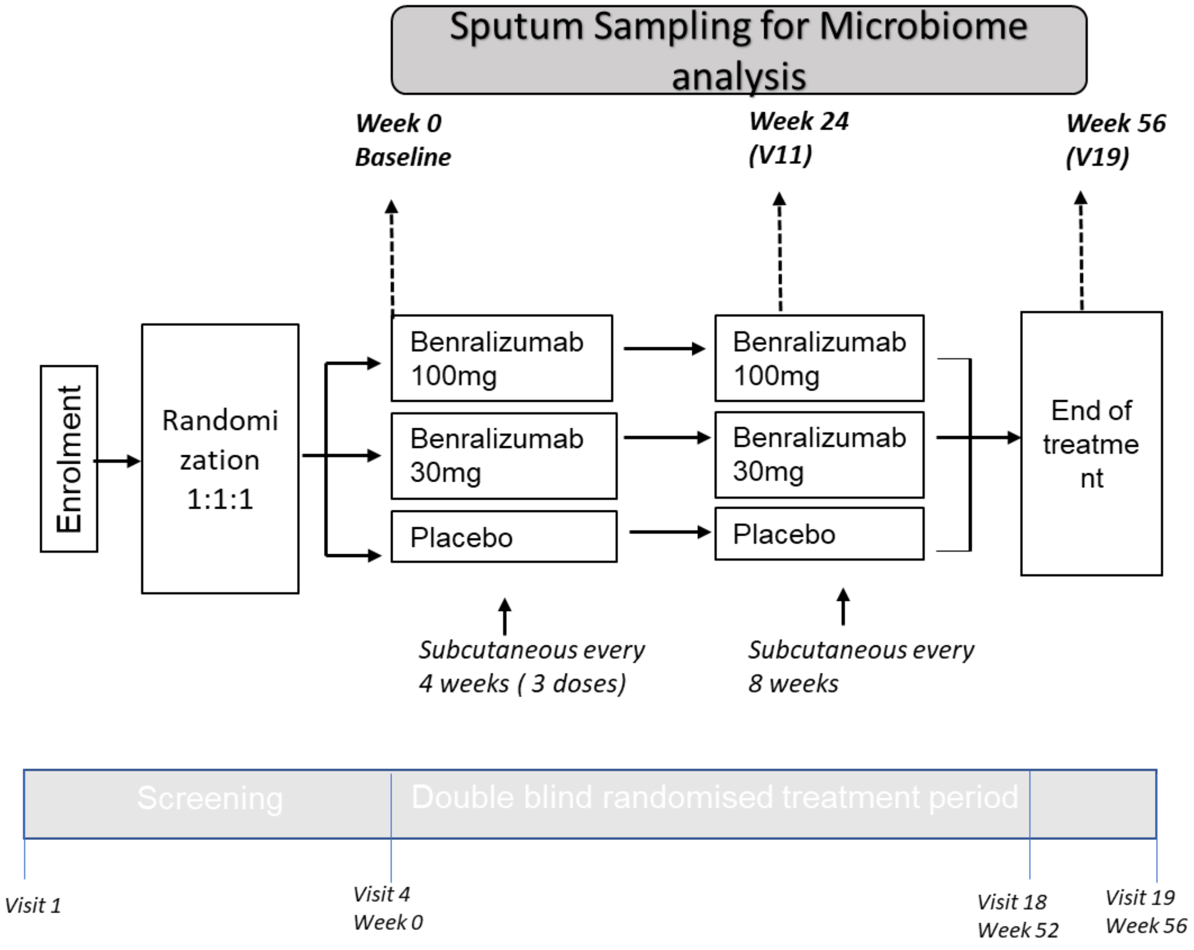

3

4 **Supplementary fig 1: GALATHEA trial study design**

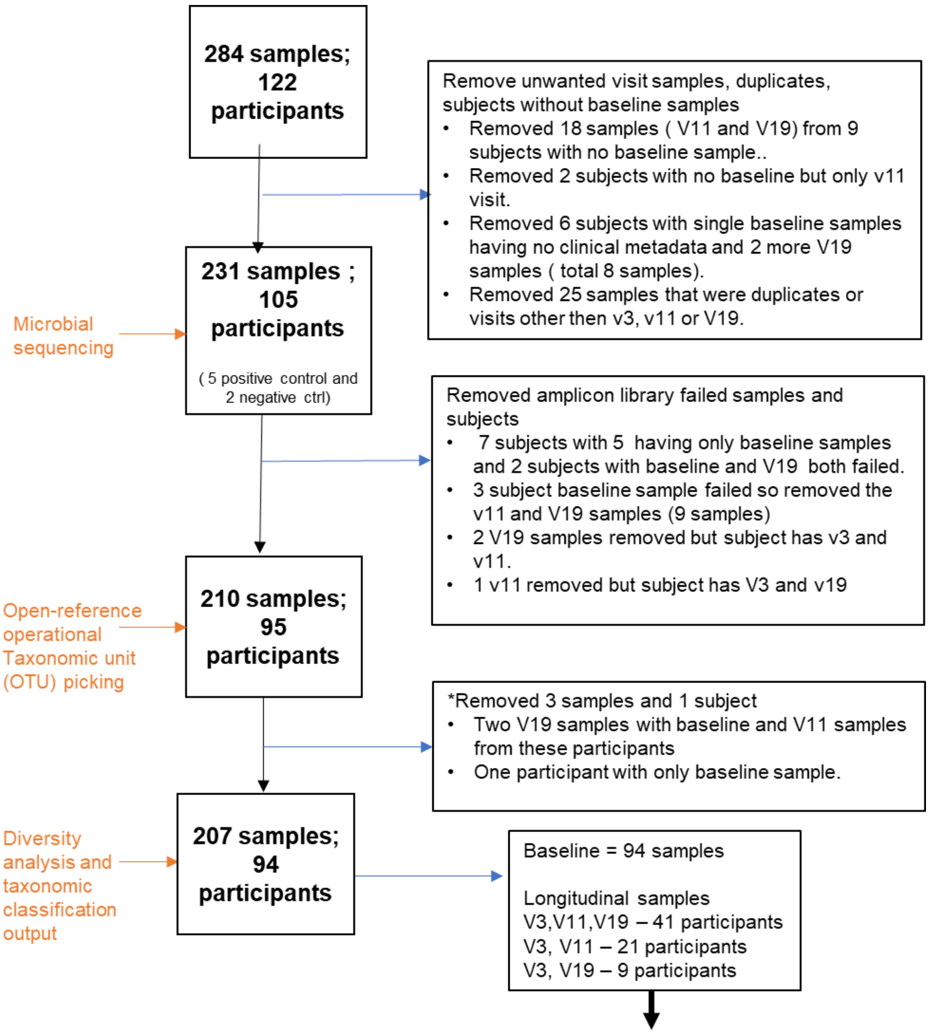

Longitudinal samples by treatment	No of visits	No of samples	subjects
Benra 100mg	2	18	9
	3	30	10
Benra 30mg	2	14	7
	3	48	16
Placebo	2	28	14
	3	45	15

Supplementary fig 2. Participants and samples included for final microbiome analysis

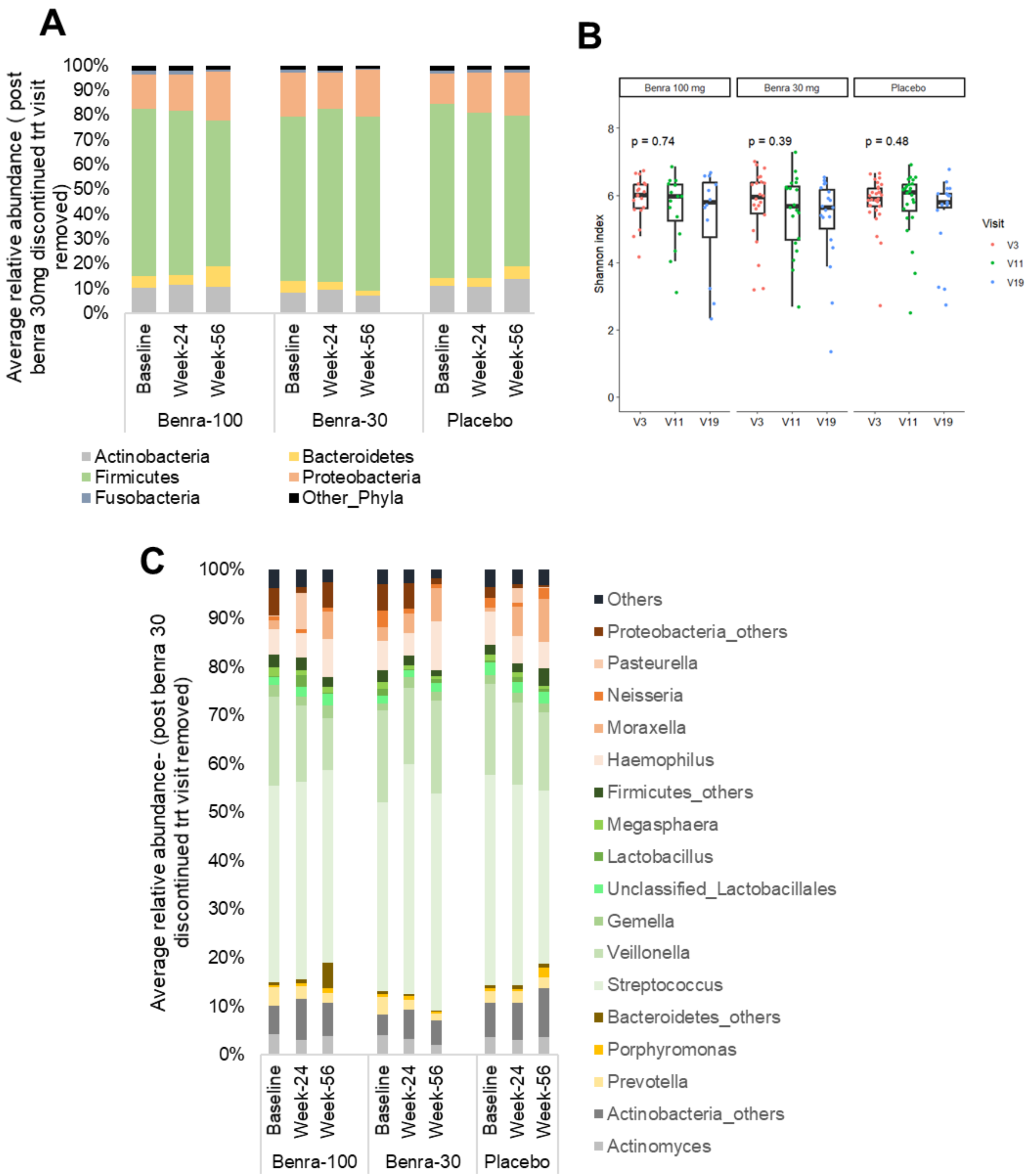

Supplementary fig 3. No change in microbiome with removal of post treatment samples from discontinued treatment participants A and C represents the taxonomic distribution at phylum and genus level; B represents the alpha diversity

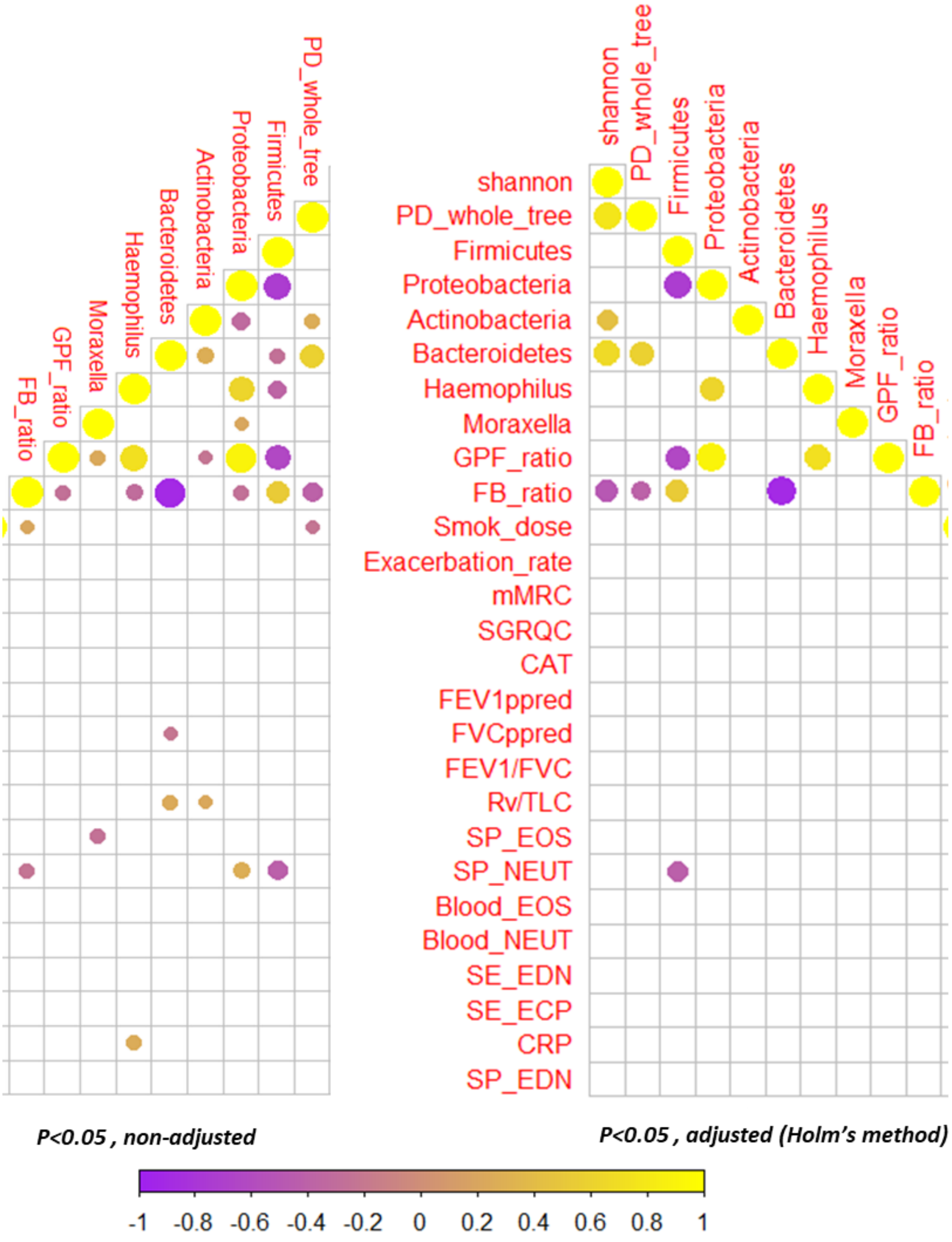

12 **Supplementary fig 4. Baseline association of microbiome parameters with**
13 **clinical features and inflammatory biomarkers** the size and the colour intensity of
14 the dot are proportional to the correlation coefficient. Colour scale shows the
15 correlation coefficients. Empty columns didn't show significant correlation

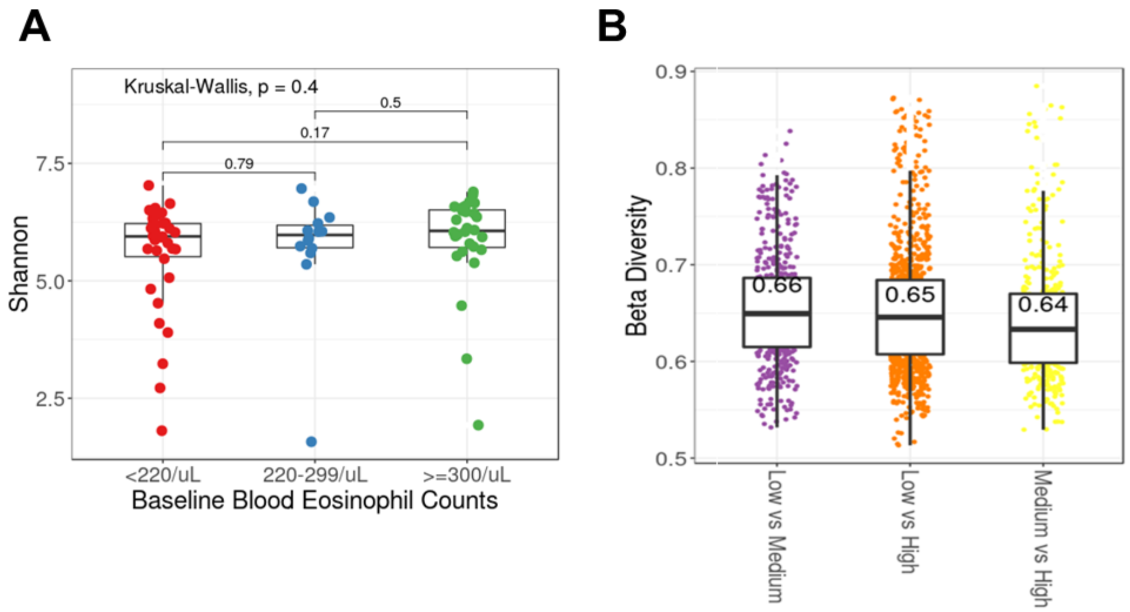

17 **Supplementary fig 5. No difference in diversity metrics between the baseline blood eosinophil groups** A represents the alpha diversity and B represents the beta diversity of baseline samples between the three eosinophil groups

	**Benralizumab 100mg**						**Benralizumab 30mg**					
	**Week 24**			**Week 56**			**Week 24**			**Week 56**		
Change from baseline	Estimate	Std error	p	Estimate	Std error	p	Estimate	Std error	p	Estimate	Std error	p
Blood eosinophils	-3.05	0.21	**6E-50**	-3.28	0.20	**8E-58**	-2.26	0.31	**1E-13**	-2.58	0.36	**5E-13**
Sputum eosinophils	-1.03	0.51	**0.04**	-0.99	0.69	**0.15**	-1.66	0.72	**0.02**	-1.66	0.99	**0.09**
Serum EDN	-1.54	0.12	**5E-11**	-1.79	0.19	**5E-09**	-1.42	0.18	**8E-09**	-1.18	0.25	**3E-05**
Serum ECP	-0.95	0.12	**1E-08**	-0.97	0.13	**6E-08**	-0.92	0.15	**9E-07**	-1.11	0.16	**9E-08**
Sputum EDN	-2.04	0.77	**0.02**	-2.07	1.05	0.06	-2.40	0.82	**0.01**	-2.25	1.23	0.08

	**Placebo**					
	**Week 24**			**Week 56**		
Change form Baseline	Estimate	Std error	p	Estimate	Std error	p
Blood eosinophils	-0.25	0.11	0.03	-0.31	0.14	0.03
Sputum eosinophils	-0.08	0.28	0.77	-0.17	0.28	0.55
Serum EDN	-0.02	0.01	0.05	-0.04	0.02	0.03
Serum ECP	-0.26	0.12	0.03	-0.26	0.13	0.06
Sputum EDN	-0.98	0.67	0.16	-0.14	0.96	0.88

Supplementary table 1. Benralizumab treated samples showed significant depletion in eosinophil markers at week 24 and week 56 Linear mixed model was used to assess within treatment group differences. Baseline time point were set as the reference group. Estimates provide the estimated value of drop in immune markers from baseline.

A

Change from placebo	V11:Benralizumab 100mg			V19:Benralizumab 100mg		
	Estimate	Std error	p	Estimate	Std error	p
CAT	-1.19	1.68	0.48	-3.97	1.84	**0.03**
Blood eosinophils	-2.04	0.55	**2.1E-04**	-2.07	0.56	**1.9E-04**
Sputum eosinophils	-1.08	0.65	0.10	-0.80	0.80	0.32
Serum EDN	-1.39	0.17	**1.1E-11**	-1.51	0.26	**1.9E-07**
Serum ECP	-0.68	0.19	**3.9E-04**	-0.71	0.21	**1.0E-03**
	**V11:Benralizumab 30mg**			**V19:Benralizumab 30mg**		
CAT	-0.30	1.58	0.85	-1.07	1.68	0.53
Blood eosinophils,	-1.46	0.35	**2.5E-05**	-1.84	0.43	**1.8E-05**
Sputum eosinophils	-1.93	0.93	**0.04**	-2.35	1.15	**0.04**
Serum EDN	-1.27	0.17	**1.1E-10**	-0.82	0.25	**1.7E-03**
Serum ECP	-0.65	0.18	**3.7E-04**	-0.83	0.20	**5.3E-05**

B

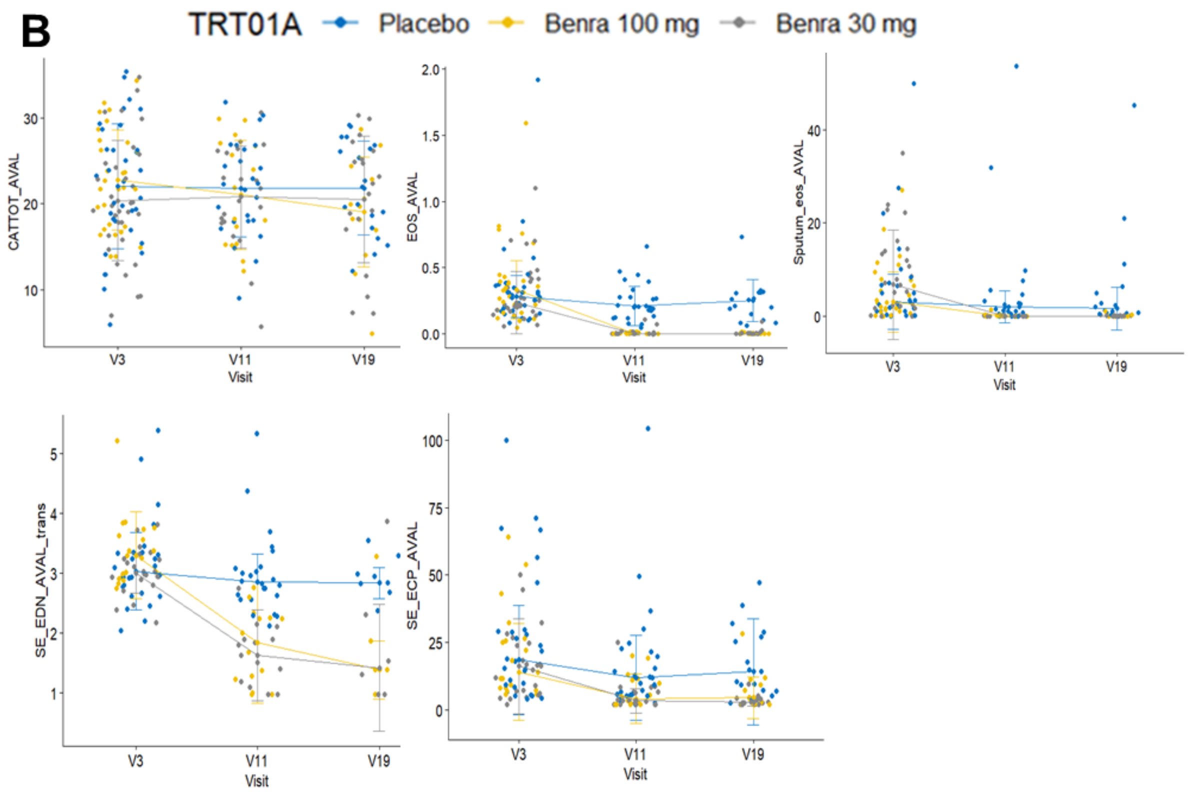

Supplementary fig 6. Benralizumab treated samples showed significantly lower eosinophil markers at week 24 and week 56 compared to placebo Linear mixed model was used to assess the effect of interaction between treatment and visit timepoint. Placebo and baseline time point were set as the reference group. Subjects were included as random factor. **A** shows the estimated values based on linear

model; **B** represents the median (IQR) across the 3 visit timepoint in each of the treatment groups.

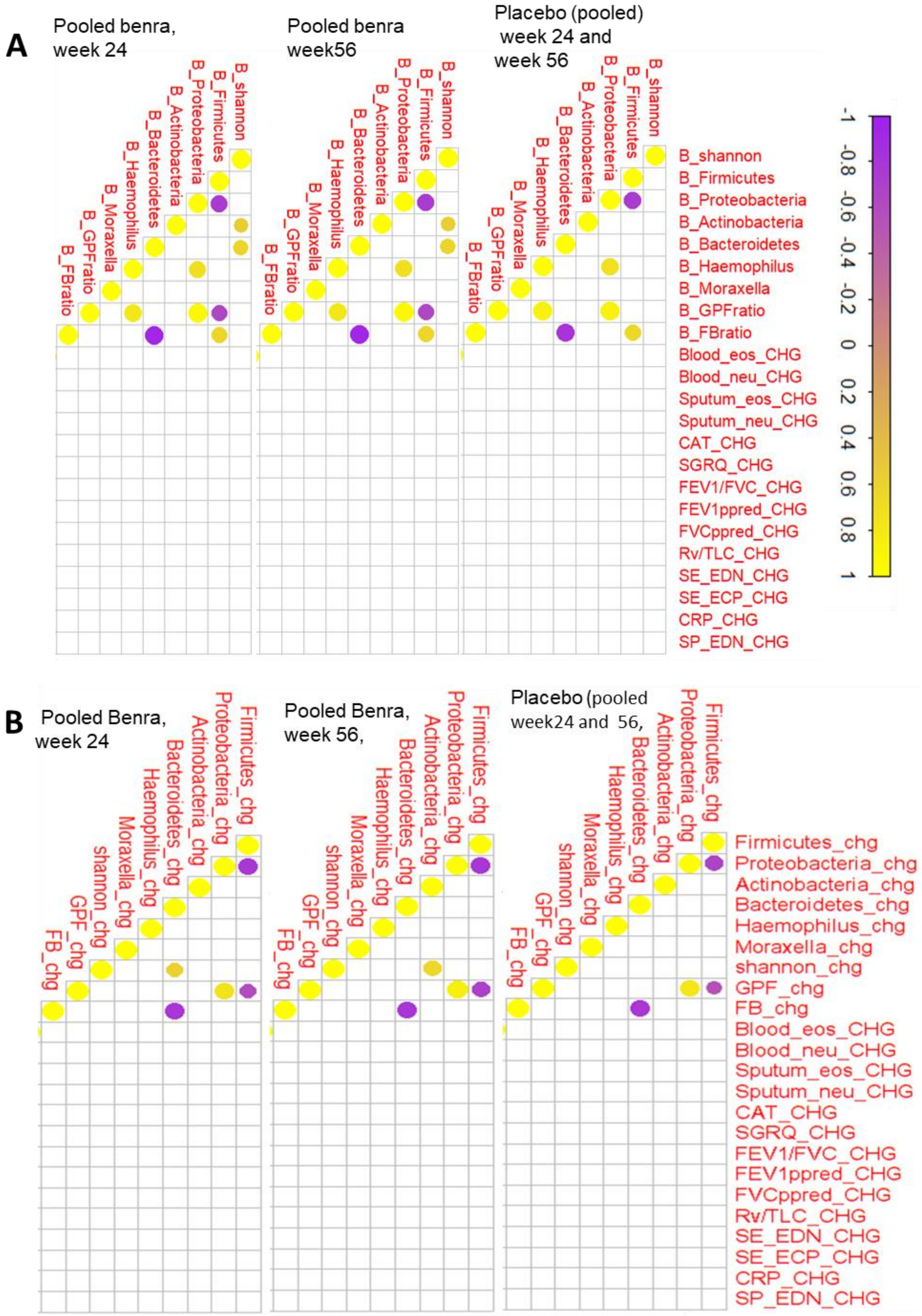

Supplemantary fig 7 A. Correlation of baseline microbiome parameters with change in clinical features B. Correlation between change in microbiome

parameters and change in clinical features Only significant and multiple comparison corrected correlation coefficient are represented in both A and B
